# What’s next for WHO’s global strategy to reduce the harmful use of alcohol?

**DOI:** 10.2471/BLT.19.241737

**Published:** 2020-01-28

**Authors:** David H Jernigan, Pamela J Trangenstein

**Affiliations:** a Boston University School of Public Health, Department of Health Law, Policy, and Management, 715 Albany St., Boston, MA 02118, United States of America (USA).; b Department of Health Behavior, University of North Carolina at Chapel Hill Gillings School of Global Public Health, Chapel Hill, USA.

Almost 10 years after the World Health Assembly adopted the *Global strategy to reduce harmful use of alcohol*, and seven years after the inclusion of alcohol as one of the key risk factors in the World Health Organization’s (WHO’s) *Global action plan for the prevention and control of noncommunicable diseases 2013–2030,* Member States have made little progress in addressing alcohol use as a risk factor for health.

We reach this conclusion based on analysis of Member States’ self-reports of actions to reduce the harmful use of alcohol. We used data on alcohol policies from two recent surveys conducted by WHO: a 2015 global questionnaire on the progress on the global alcohol strategy since 2010,^1^ answered by 138 Member States, and the 2016 Global survey on alcohol and health, answered by 173 Member States. Results were published in WHO’s 2018 *Global status report on alcohol and health*,^2^ mostly with data from the 2016 survey, but also including some data from the 2015 survey. 

Both surveys showed that progress on alcohol policies has been slow. The alcohol strategy outlined 10 areas,^3^ with three identified as the most effective and cost–effective interventions to reduce alcohol-related harm, costing less than 100 United Sates dollars (US$) per disability-adjusted life year averted. These interventions include strengthening restrictions on alcohol availability, bans or comprehensive restrictions on alcohol advertising across multiple media platforms and increasing alcohol excise taxes.

Of these three interventions, countries were most active on taxes, because most depend on tax revenues for national budgets. Member States appear unaware of taxes’ critical public health role in managing the affordability of alcohol. Most countries (155 of the 163 non-Muslim countries responding to the 2016 survey) apply sales tax to beer. The 2015 survey on policy changes revealed that 78 (62%) of the 126 countries that answered this question reported increasing alcohol taxes since 2010; however, only a third of these increases were described by Member States as substantial. In 45 (36%) of these 126 countries, taxes on beer stayed roughly the same. More than two thirds of countries (68%) with excise taxes did not adjust them for inflation, so the likely effect is that alcohol taxes, and by extension prices, have fallen because they are based on beverage volumes, which do not change.

Regarding alcohol marketing, the least restrictive policies were the most common, with small countries, globally, and countries in Africa and the Americas most likely to have no restrictions. According to the 2015 survey, since 2010, 8% (11/138) of countries reported a decrease in progress in this area, while 34% (47/138) reported an increase; 58% (80/138) stayed about the same. Smaller countries, overall, lagged behind their larger counterparts. Countries that reported increases in regulation of marketing of alcoholic beverages had on average twice as many residents as countries that reported decreases (average population of 52.9 million versus 22.5 million). Seven countries introduced a new total ban on alcohol marketing since 2010; however, there has been little regulation of new marketing techniques, an area where industry activities are quickly growing. For example, in the United States of America in 2017, according to Advertising Age, a global beer company spent nearly US$ 1 billion on unmeasured marketing including digital, nearly 60% more than it spent on the traditional measured media channels of print, radio and television.^4^

Results are worst for physical availability, where aside from minimum age purchase laws, most restrictions, such as limits on days and hours of sale, or on licenses to produce, distribute or sell alcohol, declined over time. Underscoring a key disparity, alcohol availability policies are becoming less restrictive in low-income countries.

## Growing evidence of harm

Meanwhile, evidence of the harm caused by alcohol has strengthened. Perhaps most compelling was the debunking of the benefits of moderate consumption. A recent systematic review and meta-analysis of 694 individual and population-level data sources concluded that the safest level of alcohol consumption is none.^5^ Advanced designs and more careful measurement of non-drinkers now suggest that so-called benefits may simply be an artefact of residual confounding.^6^

Recent studies have also highlighted alcohol’s role as a carcinogen,^7^ with one in eight alcohol-attributable deaths caused by cancer.^2^ Other key advances concern the harms from alcohol on non-drinkers and the role of alcohol in communicable diseases, such as tuberculosis, human immunodeficiency virus and other sexually transmitted infections. Alcohol use can play both a biological role (affecting immunity) and a social one (impaired decision-making and poor adherence to treatment protocols) in communicable disease transmission and outcomes.^8^^,^^9^ These new data have not yet been fully integrated into global strategies and action plans.

## Barriers to action

Meanwhile, countries consistently report barriers to effective action. Minimal resources have been devoted to implementing the global alcohol strategy at WHO’s headquarters or regional offices, resulting in insufficient technical assistance to support meaningful action. In the open-ended questions at the end of the 2015 survey, 15 countries out of 138 reported lack of coordination, including absence of a coordinating agency, and nine reported lack of data and monitoring systems. In locations without such leadership, coordination and systems, the most effective interventions, which are often technically complex, can be difficult to implement. Several countries (10 out of 138) mentioned interference by the alcohol industry. The interventions generally face significant opposition from the alcoholic beverages industry, as evidenced by the industry’s years of opposition to Scotland’s ultimately successful efforts to implement minimum unit pricing.

In its *Global action plan for the prevention and control of noncommunicable diseases 2013–2030*, WHO set a goal of reducing harmful use of alcohol by 10% by 2025. We agree with other researchers’ predictions^10^^,^^11^ that it is unlikely that Member States will achieve this goal. Per capita consumption of alcohol is one indicator used to assess progress towards this goal, yet leading alcohol epidemiologists recently concluded that global per capita consumption among adults (aged 15 years or older) would likely increase from 6.5 litres in 2017 to 7.6 litres by 2030 (an increase of 17%).^11^ Low- and middle-income countries are expected to contribute the largest increases in consumption.^11^

## Future directions

Alcohol continues as the seventh leading risk factor, responsible for approximately 3 million deaths per year, and the leading cause of death among persons aged 15 to 49 years. Alcohol is also, notably, the only drug for which there is no international convention.^2^^,^^10^ Along with alcohol-related harm, the alcohol industry and its marketing activities transcend national borders and call for a transnational response. Both the alcohol and the noncommunicable diseases global strategies have relied on voluntary action by Member States. Neither has shown success in stemming the rise of alcohol consumption and alcohol-related harms.

A more binding approach needs to be explored. A framework convention or comparable agreement could provide an international and legally binding treaty establishing general goals and principles for regulation while allowing individual countries to set specific standards at the national level. The Region of the Americas has already made substantial progress in articulating such principles for alcohol marketing.^12^

WHO’s development of the new SAFER alcohol control initiative is an important contribution, but will have minimal impact without a stronger global commitment. The 2016 Global survey on alcohol and health found that of the countries that answered the related question, 52% (84/162) had no regulations on alcohol marketing on the internet or on social media and 53% (86/162) no regulations on days of sale for beer (71%, or 113/159, off-premise, and 74% or 118/160 on-premise), or on number and density of alcohol outlets (73% or 116/158, on-premise, and 80% or 127/158 off-premise).^2^

This situation shows the need for a stronger global instrument that would allow countries to move forward together, which should be accompanied by substantially greater resources at the regional and global levels. Calls for such a framework convention on alcohol have come recently from researchers,^10^ as well as the World Medical Association, the American Public Health Association and the American Society for Addiction Medicine.

We recommend that the World Health Assembly request the Director General to begin the process of investigating the need for – and feasibility of – a global legally-binding instrument to reduce alcohol-related harm. This process needs to be accompanied by enough resources to inform and equip Member States with the necessary technical public health background. The Framework Convention for Tobacco Control took 10 years to develop and implement, so this is likely a long-term process.^10^ The softer approach of two global strategies targeting alcohol-related harm has been tried; it is time to explore more effective avenues of global action.

## References

[1] Jernigan DH, Trangenstein P. Global developments in alcohol policies: progress in implementation of the WHO Global strategy to reduce harmful use of alcohol since 2010. Geneva: World Health Organization; 2016 [cited 2019 July 20]. Available from: Available https://www.who.int/substance_abuse/activities/fadab/msb_adab_gas_progress_report.pdf?ua=1

[2] Global status report on alcohol and health 2018. Geneva: World Health Organization; 2018. Available from: https://www.who.int/substance_abuse/publications/global_alcohol_report/en [cited 2019 Jul 20].

[3] Global strategy to reduce the harmful use of alcohol. Geneva: World Health Organization; 2010. Available from: http://www.who.int/substance_abuse/alcstratenglishfinal.pdf?ua=1 [cited 2015 April 2].

[4] Age-Neustar A. (2018). 200 Leading National Advertisers 2018 Fact Pack. Available from: http://adage.com/d/resources/system/files/resource/Neustar-2018%20LNA%20Fact%20Pack.pdf [cited 2020 Feb 17].

[5] GBD 2016 Alcohol Collaborators. Alcohol use and burden for 195 countries and territories, 1990-2016: a systematic analysis for the Global Burden of Disease Study 2016. Lancet. 2018 9 22;392(10152):1015–35. 10.1016/S0140-6736(18)31310-2 30146330 PMC6148333

[6] Andréasson S, Chikritzhs T, Dangardt F, Holder H, Naimi T, Stockwell T. Moderate alcohol consumption brings no positive effect on health. A critical research analysis. Lakartidningen. 2016 2 16;113:113. 26881794

[7] Bagnardi V, Rota M, Botteri E, Tramacere I, Islami F, Fedirko V, et al. Light alcohol drinking and cancer: a meta-analysis. Ann Oncol. 2013 2;24(2):301–8. 10.1093/annonc/mds337 22910838

[8] Parry C, Rehm J, Poznyak V, Room R. Alcohol and infectious diseases: an overlooked causal linkage? Addiction. 2009 3;104(3):331–2. 10.1111/j.1360-0443.2008.02500.x 19207335

[9] Berry MS, Johnson MW. Does being drunk or high cause HIV sexual risk behavior? A systematic review of drug administration studies. Pharmacol Biochem Behav. 2018 1;164:125–38. 10.1016/j.pbb.2017.08.009 28843425 PMC5747990

[10] Au Yeung SL, Lam TH. Unite for a framework convention for alcohol control. Lancet. 2019 5 4;393(10183):1778–9. 10.1016/S0140-6736(18)32214-1 30955974

[11] Manthey J, Shield KD, Rylett M, Hasan OSM, Probst C, Rehm J. Global alcohol exposure between 1990 and 2017 and forecasts until 2030: a modelling study. Lancet. 2019 6 22;393(10190):2493–502. 10.1016/S0140-6736(18)32744-2 31076174

[12] Technical note: Background on alcohol marketing regulation and monitoring for the protection of public health. Washington, DC: Pan American Health Organization; 2017. Available from: http://iris.paho.org/xmlui/handle/123456789/33972 [cited year month day].

